# Unique Presentation of Microscopic Polyangiitis: Hearing and Vision Loss, Dysphagia, and Renal Dysfunction

**DOI:** 10.7759/cureus.14069

**Published:** 2021-03-23

**Authors:** Rohit R Badia, Allen R Hendricks, Carlos L Perez, Anthony Sertich, Lindsay Ripley

**Affiliations:** 1 Department of Internal Medicine, University of Texas Southwestern Medical Center, Dallas, USA; 2 Department of Pathology, University of Texas Southwestern Medical Center, Dallas, USA; 3 Department of Radiology, University of Texas Southwestern Medical Center, Dallas, USA

**Keywords:** autoimmune vasculitis, microscopic polyangiitis, anti-neutrophil cytoplasmic autoantibodies, necrotizing and crescentic glomerulonephritis, dysphagia, vision loss

## Abstract

Microscopic polyangiitis (MPA) is an autoimmune small-vessel vasculitis often positive for perinuclear anti-neutrophil cytoplasmic antibody (p-ANCA), or anti-myeloperoxidase (MPO), that classically affects the lungs, kidneys, and skin. Several atypical presentations of MPA involving other organs have also been reported in the literature. We report a unique case of a patient who presented with rare presentations of MPA: hearing and vision loss, dysphagia, renal dysfunction. Despite the atypical nature of her symptoms, her p-ANCA serology was positive and kidney biopsy was consistent with MPA. Regardless of the bizarre nature of a patient’s symptoms, we highlight the importance of considering MPA as a differential diagnosis in the setting of positive p-ANCA serology.

## Introduction

Microscopic polyangiitis (MPA) is an autoimmune small-vessel vasculitis that classically has positive perinuclear anti-neutrophil cytoplasmic antibody (p-ANCA) serology and presents with hemoptysis, rapidly progressive glomerulonephritis, and palpable purpura. In the United States, the prevalence of this disease is 1.3 per 100,000 persons and typically affects middle-aged Caucasian men or women equally [1]. Though renal symptoms predominate in patients with this disease, presentations of MPA affecting other organs have been described [2-4].

Though the sensitivity and specificity of p-ANCA in diagnosing MPA approaches 90%, the diagnosis must be made based on histology [5]. We report a unique case of a patient with positive p-ANCA serology presenting with atypical presentations of MPA affecting her hearing, vision, esophageal motility, and kidneys. To our knowledge, this is the first case of a patient with biopsy-confirmed MPA with such multiple symptoms.

## Case presentation

The patient is a 64-year-old Caucasian woman with a past medical history of hypothyroidism and hypersensitivity pneumonitis who presented to the hospital with sudden near total right-sided vision loss. Six months prior to presentation, the patient developed moderate bilateral sensorineural hearing loss, which did not subside despite two separate corticosteroid courses by an otolaryngologist. Records from the otolaryngologist were unavailable.

Two months prior to presentation, the patient developed a metallic taste in her mouth which progressed to odynophagia, culminating in decreased oral intake and a 40-pound weight loss. GI workup performed prior to presentation at our institution was ultimately inconclusive: endoscopy revealed mild esophagitis and pathology showed inflammation consistent with reflux but no metaplastic changes; barium swallow and video swallow study indicated mild esophageal dysmotility.

At admission, in addition to vision loss, the patient noted headaches, fatigue, and constipation over the last few months. She denied fevers, chills, jaw claudication, joint pain, and similar family history. On physical exam, the pertinent findings included bilateral temporal wasting and a mydriatic right pupil with a relative afferent pupillary defect. Slit-lamp exam was normal and dilated fundus exam was remarkable for dot and blot hemorrhage in the periphery of the right eye. The remainder of her exam was unremarkable.

Labs on presentation are listed in Table 1 along with labs from one month prior to admission. Pertinent labs at presentation included blood urea nitrogen (BUN) of 60 mg/dL, Creatinine (Cr) of 3.92 mg/dL, fractional excretion of sodium of 7.3%, urine protein of 23 mg/dL, urine red blood cell (RBC) count of 4 cells/hpf, white blood cell (WBC) count of 25.84 x 10^9^/L, RBC count of 3.71 x 10^9^/L, platelet count of 376 x 10^9^/L, erythrocyte sedimentation rate (ESR) of 62 mm/hr, and C-reactive protein (CRP) of 15.9 mg/dL. True baseline labs for this patient before onset of symptoms are not available.

**Table 1:** Clinical characteristics of the patient at presentation. Data of interest is bolded and unavailable data are noted with “-”. BUN - Blood urea nitrogen, Cr - Creatinine, eGFR - Estimated glomerular filtration rate, AST - Aspartate transaminase, ALT - Alanine transaminase, ALP - Alkaline phosphatase, WBC - White blood cell count, RBC - Red blood cell count, ANA - Antinuclear antibody, c-ANCA - Antineutrophil cytoplasmic antibodies, p-ANCA - Perinuclear anti-neutrophil cytoplasmic antibodies, ENA - Extractable nuclear antigen, RNP -Ribonucleoprotein, MPO - Myeloperoxidase, PR3 - Proteinase 3, ESR - Erythrocyte sedimentation rate, CRP - C-reactive protein, TSH - Thyroid-stimulating hormone

		Presentation	1 month prior
Chemistry Profile	Sodium (mEq/L)	131	134
	Potassium (mEq/L)	4.9	3.5
	Chloride (mEq/L)	97	100
	Bicarbonate (mEq/L)	21	23
	BUN (mg/dL)	60	5
	Cr (mg/dL)	3.92	0.62
	eGFR (mL/min/1.73m^2^)	12	95
	Calcium (mg/dL)	8.6	8.4
	Phosphorous (mg/dL)	4.6	3.7
Liver Profile	AST (Units/L)	20	47
	ALT (Units/L)	22	72
	ALP (Units/L)	253	461
	Total bilirubin (mg/dL)	0.5	0.5
CBC	WBC (x 10^9^/L)	25.84	13.69
	RBC (x 10^9^/L)	3.71	3.85
	Hemoglobin (g/dL)	9.8	10.3
	Platelets (x 10^9^/L)	376	486
Immunology	Rheumatoid factor (IU/mL)	۔	30
	ANA	۔	<1:40
	c-ANCA	<1:20	۔
	p-ANCA	1։80	۔
	ENA Jo-1 Ab	<0.2	۔
	ENA RNP Ab	<0.2	۔
	ENA Scl-70 Ab	<0.2	۔
	ENA Smith (Sm) Ab	<0.2	۔
	ENA SSA (Ro) Ab	<0.2	۔
	ENA SSB (La) Ab	<0.2	۔
	Anti-MPO Ab	<9.0	۔
	Anti-PR3 Ab	<3.5	۔
	Cryoglobulin	Negative	۔
Infectious Disease	SARS-CoV-2	Negative	Negative
	Blood cultures	Negative	Negative
	Urine culture	Negative	Negative
	Hepatitis A status	Negative	Negative
	Hepatitis B status	Negative	Negative
	Hepatitis C status	Resolved infection	Resolved infection
Urine	Protein (mg/dL)	23	۔
	RBC (cells/hpf)	4	1
	WBC (cells/hpf)	3	2
	Creatinine (mg/dL)	28.4	۔
	Sodium (mEq/L)	69	۔
	Protein to Creatinine Ratio	0.81	۔
Other	ESR (mm/hr)	62	83
	CRP (mg/dL)	15.9	18.15
	TSH (mcIU/mL)	3.96	2.23

**Figure 1:**
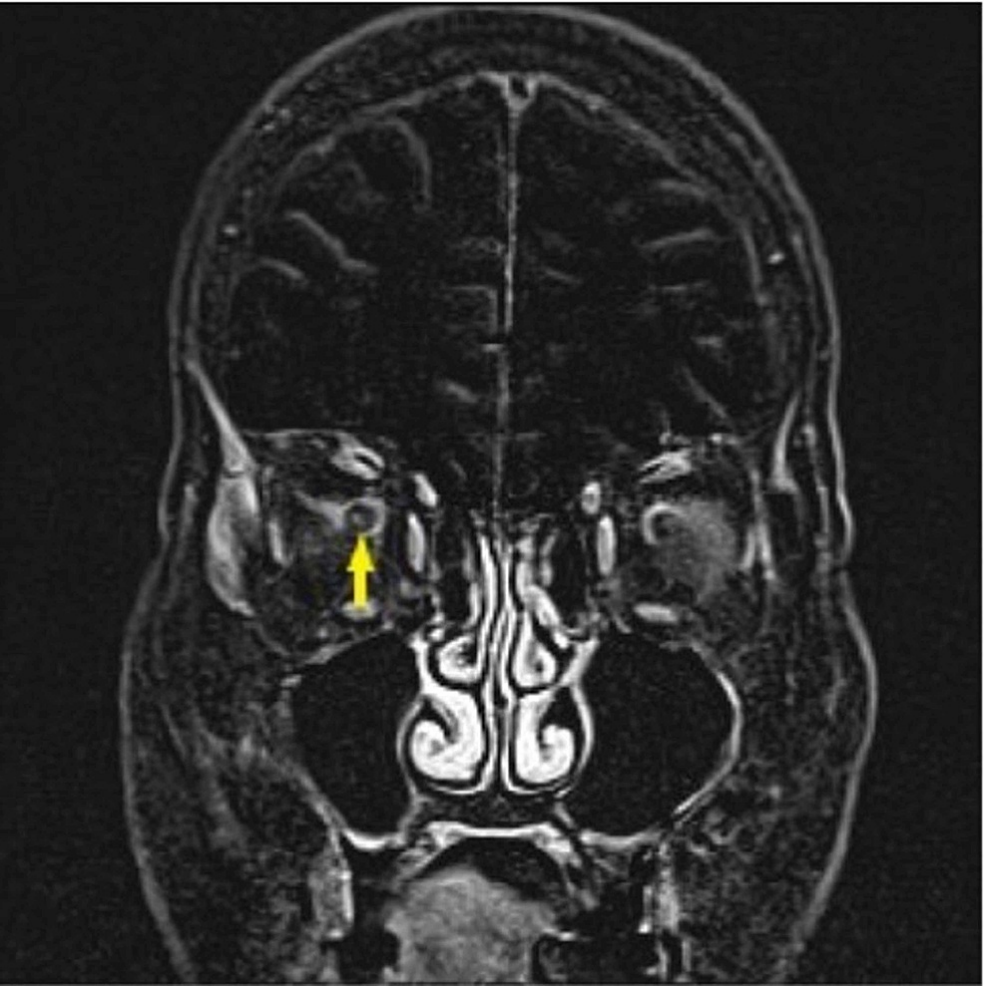
Coronal T1 post Gadolinium subtracted image demonstrates asymmetric enhancement of the right optic nerve (yellow arrow).

MRI orbits with contrast (Figure 1) were done at presentation despite the patient's abnormal renal function. Studies have shown that MRI with contrast allows for better detection of giant cell arteritis (GCA) [6]. Imaging was read as having “asymmetric increased enhancement within the right proximal optic nerve and right optic disc.” That is, there was a slightly increased signal of the right optic nerve head and proximal intraconal optic nerve compared to the left side after the administration of IV Gadolinium.

Due to suspected GCA, the patient was started on high dose IV methylprednisolone 250 mg every six hours for three days followed by oral prednisone 60 mg thereafter. The patient’s BUN and creatinine continued to increase, reaching a maximum BUN of 120 mg/dL and maximum creatinine of 4.6 mg/dL. Renal ultrasonography revealed no hydronephrosis or calculi. This raised concern for an intrarenal pathology.

**Figure 2:**
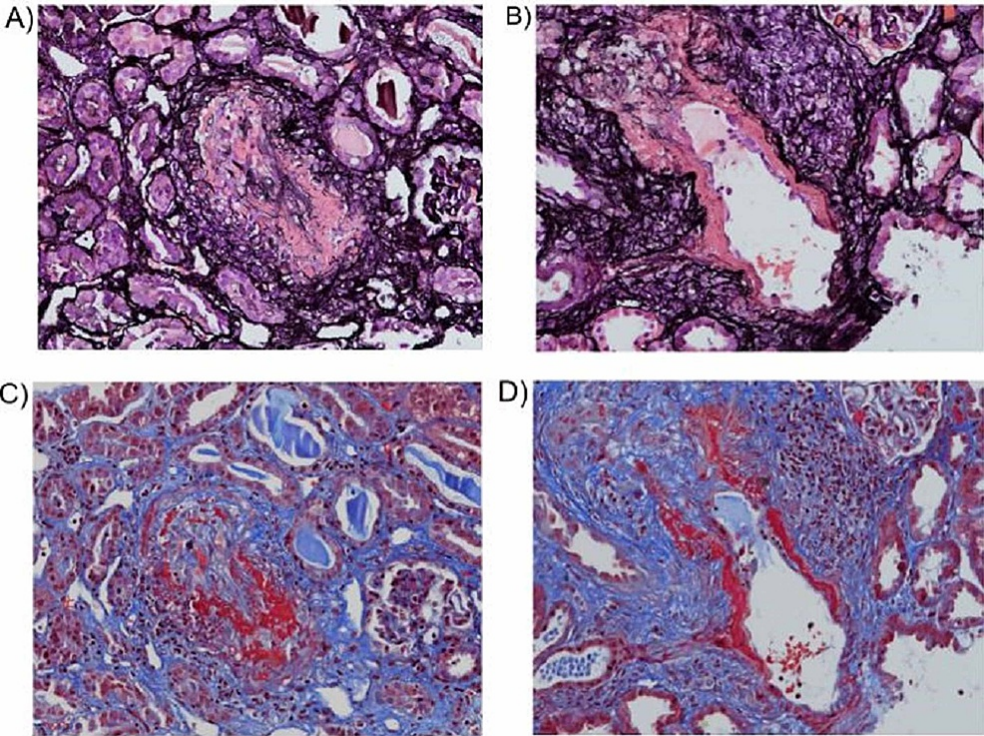
Light microscopy findings. (A, B) Jones methenamine silver stain showing extensive necrotizing arteritis affecting two interlobular arteries. The fibrinoid necrosis is highlighted in dark pink (x 200). (C, D) The same two arteries have fibrinoid necrosis highlighted in red by a Masson trichrome stain (x 200).

A kidney biopsy was performed. Light microscopy showed eighteen unremarkable glomeruli with no crescents, fibrinoid necrosis, endocapillary hypercellularity, or segmental sclerosis. There was mild (approximately 20%) interstitial fibrosis and tubular atrophy. Focal mild tubulitis was present. A patchy, mild infiltrate of mononuclear leukocytes was seen in the intact interstitium. Two interlobular arteries with necrotizing arteritis were identified (Figure 2). A third interlobular artery was infiltrated by inflammatory cells but had no necrosis. Immunofluorescence and electron microscopy showed no significant findings. The patient was also found to have positive p-ANCA and negative anti-myeloperoxidase (MPO) Ab and anti-PR3 Ab serologies. The serology and kidney biopsy findings are indicative of her recent deterioration of renal function and consistent with a diagnosis of MPA.

Given prior dysphagia work up, further studies were not pursued during this hospitalization. The patient tolerated tube feeds via Dobhoff tube, which was later replaced by a percutaneous endoscopic gastrostomy (PEG) by the gastroenterology service.

For treatment, the patient received 500 mg/m^2^ of IV cyclophosphamide and 2-mercaptoethane sulfonate sodium (MESNA) with a plan for continued treatments every two weeks for three to six months. The patient was scheduled to finish one month of therapy on Prednisone 60 mg daily, then started a prolonged taper with further course to be determined based on symptom improvement. Rituximab was also discussed as an alternative to cyclophosphamide, but the latter was chosen due to cranial nerve involvement. At discharge, the patient’s vision remained unchanged, and she was scheduled to follow up with ophthalmology.

## Discussion

MPA is a small vessel vasculitis that occurs predominantly in Caucasians over the age of 50 [1]. Patients can have systemic symptoms such as fever, weight loss, and loss of appetite [7]. MPA can cause severe organ damage, including kidney dysfunction, diffuse alveolar hemorrhage, pulmonary fibrosis, mononeuritis multiplex, and rashes including ulcers and palpable purpura [7]. Several MPA manifestations share clinical features with polyarteritis nodosa and granulomatosis with polyangiitis (GPA), so it must be distinguished based on histologic findings and serum markers [7]. For detecting MPA, p-ANCA has a sensitivity of ~87% and specificity of ~89% [5].

The patient described in this case report has demographic, histologic, and laboratory findings that are consistent with MPA. The patient is Caucasian, middle-aged, had renal insufficiency, weight loss, and a positive p-ANCA.

Hearing involvement

Despite being a demographically typical patient, the progression of symptoms was quite atypical, and we report several clinical characteristics in this patient with MPA that have not been previously published/or are minimally reported in literature. The first symptom this patient experienced is sensorineural hearing loss. Although ear, nose, and throat (ENT) involvement are common in GPA and eosinophilic GPA, data regarding the prevalence of ENT symptoms in patients MPA is varied; three separate studies have shown prevalence of 9%, 20%, and 30% [8]. In 23 patients with MPA presenting to an Otolaryngology clinic in Poland for ENT symptoms, eight patients (35%) had hearing loss [9]. A similar percentage of patients were also reported in a Japanese study [10]. Based on these statistics of prevalence of ENT symptoms in MPA patients and prevalence of hearing loss in these patients, we estimate that between 3% and 10% of patients with MPA have hearing loss.

Dysphagia

The patient next developed a metallic taste in her mouth and dysphagia. As reported earlier, pathology from endoscopic specimens was inconclusive. However, pathological findings are only present in ~5% of patients with vasculitis, so the aforementioned findings all raise concern for a vasculitis induced esophageal motility issue [11]. To our knowledge, this is one of few cases of a patient presenting with esophageal dysmotility in the setting of MPA [3].

Vision loss

This patient also developed sudden vision loss, which per the MRI orbit was concerning for acute anterior ischemic optic neuropathy and was treated as GCA. Due to the diagnosis of MPA, ophthalmology attributed the vision loss to small vessel vasculitis instead. However, there have been 15 reported cases of concurrent GCA (a large vessel vasculitis) in MPA in the literature [4]. Temporal artery biopsy was not performed, so we do not have a pathological verification for the cause of her vision loss.

Acute renal failure

The patient had an atypical presentation of acute renal failure. Although the kidneys are affected in a majority of patients with MPA, in most cases there is a greater level of proteinuria, hematuria, and/or active sediment [12]. In this patient, this was not observed. Based solely on the patient’s unremarkable urinary sediment and low proteinuria but worsening renal function, the presentation would be more consistent with a middle- or large-vessel vasculitis rather than a small-vessel vasculitis like MPA. However, her positive p-ANCA and histopathologic finding of necrotizing arteritis are diagnostic of MPA. Other potential causes of necrotizing arteritis include polyarteritis nodosa, GPA, or cryoglobulinemic vasculitis. Hence, in the setting of an atypical presentation of acute renal failure, MPA must still be considered in the differential diagnosis.

## Conclusions

The patient described in this case report had several sensory deficits caused by her illness. Her sense of sight, hearing, and taste were all impaired. We attribute all the patient’s symptoms to MPA due to her serologies (positive p-ANCA) and kidney biopsy findings (necrotizing arteritis), but it is possible that her visual deficit, auditory deficit, dysgeusia, dysphagia, and acute renal failure may be due to several distinct etiologies. In addition, because the patient had been relatively healthy until six months prior to presentation and developed multiple health problems within a short period, we believe the possibility of several unrelated conditions is remote. 

As stated earlier, p-ANCA is a highly sensitive and specific biomarker for MPA. Therefore, in the setting of positive p-ANCA serology, MPA must be considered on the differential diagnosis despite the symptoms or urinalysis findings a patient manifests. Ultimately, the diagnosis must be confirmed with a biopsy of the affected organ.
